# The Sex of Donor and Recipients in Solid Organ Transplantation: An in Depth Analysis Across the Council of Europe Member States

**DOI:** 10.3389/ti.2026.15711

**Published:** 2026-05-07

**Authors:** Emanuele Cozzi, Claudia Carella, Francesca Puoti, Lucia Masiero, John Forsythe, Derek Manas, Naomi Nathan, Artur Kaminski, Marina Álvarez, Mar Carmona, Beatriz Mahíllo, Corinne Antoine, Marta López-Fraga, Giuseppe Feltrin, Beatriz Domínguez-Gil

**Affiliations:** 1 Centro Nazionale Trapianti-Istituto Superiore di Sanità (CNT-ISS), Rome, Italy; 2 Department of Cardiac, Thoracic and Vascular Sciences, Transplant Immunology Unit, Padua University Hospital, University of Padua, Padua, Italy; 3 NHS Blood and Transplant (NHSBT), Bristol, United Kingdom; 4 Nederlandse Transplantatie Stichting, Leiden, Netherlands; 5 Department of Transplantology and Central Tissue Bank, Medical University of Warsaw, Polish Transplant Coordinating Centre POLTRANSPLANT, Warsaw, Poland; 6 Organización Nacional de Trasplantes (ONT), Madrid, Spain; 7 Agence de la Biomédecine, Saint-Denis de la Plaine, France; 8 European Directorate for the Quality of Medicines and Healthcare (EDQM), Council of Europe, Strasbourg, France

**Keywords:** council of europe, equity, organ donation, sex, transplantation

## Abstract

Sex equity in organ donation and access to transplantation represents a key priority of the European Committee on Organ Transplantation of the Council of Europe (CD-P-TO). To increase our knowledge on sex-related differences in transplantation in the Council of Europe Member States, a specifically designed questionnaire was distributed to the CD-P-TO countries. Results confirm that, irrespective of the organ, males represent the majority of patients on the transplant waiting list. For all organs except for heart the time spent on the waiting list was shorter for men compared to women. Women represent the majority of living kidney donors (58%), whilst males are the major source of livers from living donation (54%). Across all organ types, men received 64% of deceased donor organs and 58% of living donor organs. We have found sex-related differences in transplantation activities conducted in the Council of Europe Member States. However, these may be the consequence of the higher incidence of some diseases in men, organ size mismatch, or the greater difficulty in finding immunologically compatible donors in women. At this stage, the CD-P-TO will continue its monitoring activity on this highly relevant topic and possibly extend its commitment beyond sex to include gender related aspects.

## Graphical Abstract

**Figure:**
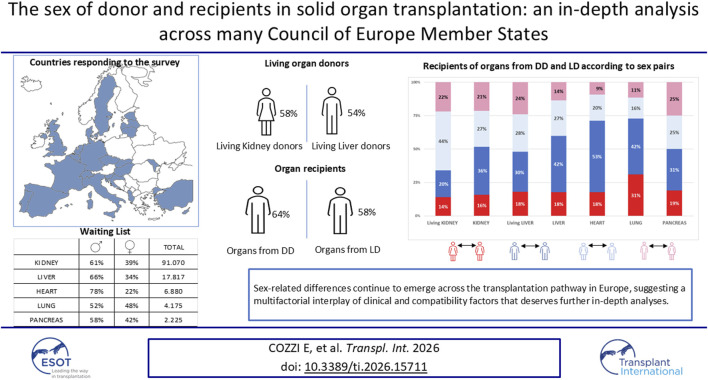

## Introduction

Gender and sex equity in organ donation and access to transplantation represent a key priority of the European Committee on Organ Transplantation of the Council of Europe (CD-P-TO). To date, the sex of donors and recipients involved in donation and transplantation activities across the Council of Europe Member States has been preliminarily investigated through the data collection undertaken by the Newsletter Transplant of the Council of Europe and a preliminary analysis on the *European landscape of donors and recipients sex in solid organ transplantation* was published in late 2022 [1].

According to our earlier international investigation involving 69 countries from four continents, globally men are the more frequent deceased donors, while women are the main source of kidneys and livers from living donors [1]. Furthermore, men have a greater access to transplantation compared to women. At present there is no reason to believe that, as far as the Council of Europe Member States, such differences between sex in organ donation and transplantation may be the ultimate outcome of an unfair process. However, some have previously reported that some of the differences could be the result of social roles or pressures on women [2]. Other studies involving men and women who served as living kidney donors did not demonstrate differences in psychosocial profiles or greater vulnerability to family pressure between sex [3]. In any case, men receive the majority of transplanted organs [4] in part because of a higher incidence of chronic diseases [5, 6] and because immunological factors may disadvantage women [7]. In addition, cultural and social factors, physician biases in the perception of patient frailty, as well as potential sex-based inequities of formulas used for organ allocation and transplant criteria may limit transplant opportunities for women [2, 8–12]. At this stage, the CD-P-TO felt that a more in depth collection and analysis of data disaggregated by sex would enable a better comprehension of the possible impact of sex and gender on transplant opportunities. To this end, the CD-P-TO prepared a specifically designed questionnaire aimed at supplementing the information already available through the Newsletter Transplant [13]. Such a questionnaire was applied to all CD-P-TO Member States and a detailed analysis of the new set of data collected is here provided.

## Materials and Methods

The aim of the survey was to collect comprehensive data on organ donors and recipients in the CD-P-TO Member States, with a particular focus on sex. The key components of the study were composition of waiting lists, donor and recipient baseline information, sex combinations of donor-recipient pairs for various organ transplants, and the 5-years graft survival rates based on these combinations. The collated dataset refers to the year 2019, which is the latest year that was not affected by the SARS-CoV-2 pandemic.

The CD-P-TO established an *ad hoc* working group lead by the Italian National Transplant Centre (CNT), who prepared a questionnaire whose final version was agreed by the CD-P-TO on 7 April 2022, and formally approved in its plenary meeting held in Warsaw on 7 October 2022. The approved version of the questionnaire is appended (Supplementary Material). The questionnaire was circulated to the 39 CD-P-TO Member States and 5 Observers Countries, and was completed by national focal points already designated by the Ministries of Health at each country for the routine data collection performed for the Newsletter Transplant. CNT then compiled the information collected by the questionnaires, assessed the accuracy and level of completeness of the data provided and performed the data analysis. In cases of inconsistencies, the CNT contacted the designated focal point in each country for a final data check.

A total number of 33 countries filled in questionnaires were received. Four member states were removed from the analysis due to incomplete data (notwithstanding the extensive efforts put in place to clarify some inaccuracies or incomplete answers). Answers to questionnaires included in the analysis were provided by 6 Ministries of Health and 23 National Competent Authorities [Supplementary Table S1]. The 5-year organ survival calculation was derived from data supplied from countries with 10 transplants or more per organ type (from either living or deceased donor).

### Waiting Lists

In the first instance we collected data on the number of male (M) and female (F) patients that were ever active on waiting lists (WL) in the year 2019. Additionally, each organ type respondents were asked to provide, when available, the median waiting time and the min-max range for all the patients on the WL, as well as the data disaggregated per male and female, respectively. The waiting time for kidney patients on the WL was calculated from the first day on dialysis. All the countries were also asked to provide both the median waiting time of any patient on the WL in 2019 irrespective as to whether the patient got transplanted or not, and the waiting time of only those who were actually transplanted in 2019.

### Donors’ Information

The questions relating to deceased organ donors focused on both donation after brain death (DBD) and donation after the circulatory determination of death (DCD), including uncontrolled and controlled DCD, namely type II, III and IV DCD Maastricht categories. For each of these, mean age and min-man range of M and F donors was collected. The same set of data was collected for living donors (LD), namely living kidney donors (LKD) and living liver donors (LLD), respectively.

### Recipients’ Information

In the section relating to recipients of solid organ transplants, countries provided the total number of M and F recipients of kidney, liver, heart, lung and pancreas from both DBD and DCD donors. The information regarding the recipients of organs from living donors in each respondent country was obtained from Newsletter Transplant.

### Transplantation and Sex Pairs

Sex pairs in organ transplantation from deceased and living donors were also investigated. For each solid organ, countries were asked to provide the total number of transplants performed according to the following sex pairs: male to male (M-M), male to female (M-F), female to female (F-F) and female to male (F-M). Additionally, for each organ, countries were asked to provide the 5 year non-censored for death graft survival according to sex pair combinations.

### Relationship Between Living Donors and Recipients

The survey addressed also the relationship between LKD and LLD and their recipients. In case of LKD and LLD between parent and child, the possible combinations were the following: mother-to-son, mother-to-daughter, father-to-son, father-to-daughter, son-to-mother, son-to-father, daughter-to-mother and daughter-to-father. In case of siblings, the possible combinations were the following: brother-to-brother, brother-to-sister, sister-to-brother and sister-to-sister. In the case of living organ donation between spouses, the combinations were husband-to-wife and wife-to-husband. Additional combinations namely husband-to-husband and wife-to-wife were provided by the respondent countries, when available.

### Statistical Analysis

When examining the sex differences, all the analyses were conducted by organ and according to participating country, and the results were compared with the mean values obtained when combining all the countries: i) number and percentages of men and women were presented in the following analyses: (a) patients enrolled in the organ WL, (b) stratified by living and deceased transplanted patients and (c) DBD and DCD donors; ii) median age of patients on the WL and of transplanted patients; iii) 5-year non-censored for death Kaplan-Meier survival probability estimates. Specific comparisons of sexes were presented according to different Living Donor-Transplanted pairs. For all statistical analyses, we used Stata version 17.0 (StataCorp, College Station, TX, USA).

## Results

A total of 29 countries accurately completed the questionnaire and were included in the analysis. These consisted of 26 CD-P-TO Member States (Albania, Austria, Belgium, Croatia, Cyprus, Czech Republic, Denmark, Estonia, France, Germany, Hungary, Ireland, Italy, Latvia, Lithuania, Luxembourg, Moldova, the Netherlands, Portugal, Slovak Republic, Slovenia, Spain, Sweden, Switzerland, Turkey and the United Kingdom) and 3 observer countries (Armenia, Georgia and Israel). The two main CD-P-TO observer countries, namely United States and Canada, could not take part to the analysis. Furthermore, the amount of data required and the degree of details requested did not allow several Member States to participate in this analysis.

### Waiting Lists

**FIGURE 1:**
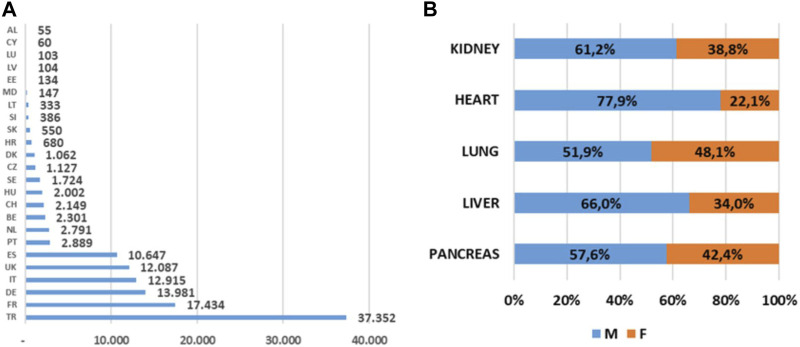
Patients on the waiting list in the CD-P-TO MS. **(A)** Total number of patients on the waiting list (any organ) at any time in 2019; **(B)** Sex distribution of patients on the waiting list in 2019 divided by organs.

**FIGURE 2:**
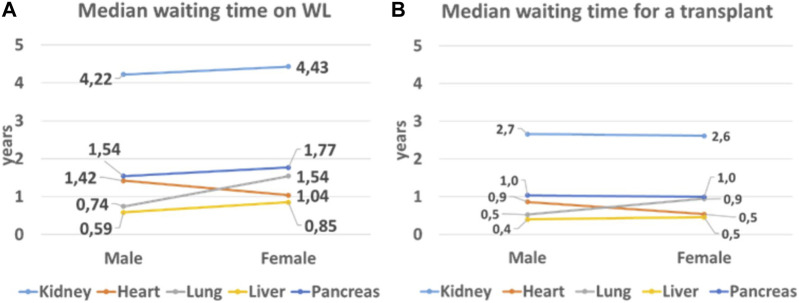
Median waiting time of patients on the WL in the CD-P-TO MS. **(A)** Waiting time of any patient on the WL in 2019 irrespective as to whether the patient got transplanted or not; **(B)** waiting time of patients who were transplanted in 2019.

For the year 2019, a total number of 122.197 patients were ever active on the WL in Member States that took part to the survey [Figure 1]. The patient distribution per organ was 91,070 for kidney (61% M; 39% F); 17,817 liver (66% M; 34% F); 6,880 heart (78% M; 22,1% F); 4,175 lung (52% M; 48% F); and 2,255 pancreas (58% M; 42% F). For each organ, the median time spent on the WL is shown in Figure 2.

### Donor Information

Transplantation after DBD donation was performed in 26 of the responding countries. Overall, 8,891 actual DBD donors were reported, of which 8,495 resulted in at least one organ transplant. The mean age of DBD donors was 54.4 years for males and 50.3 years for females. Male donors accounted for 56% of both actual and utilized DBD donors.

Only a limited number of countries performed DCD donation activities. Among the countries participating in this analysis, DCD was performed in Austria, Belgium, France, Ireland, Italy, the Netherlands, Portugal, Spain, Sweden, Switzerland, the United Kingdom and Israel. In this context, category III DCD was the most prevalent form, being performed in 12 of the respondent countries. Overall there were 1,995 actual DCD donors of which 1,745 were utilised. The mean age of category III DCD donors was 56.6 years for males and 53 years for females. Male donors accounted for 67% of actual DCD donors and 66% of utilized DCD donors.

**FIGURE 3:**
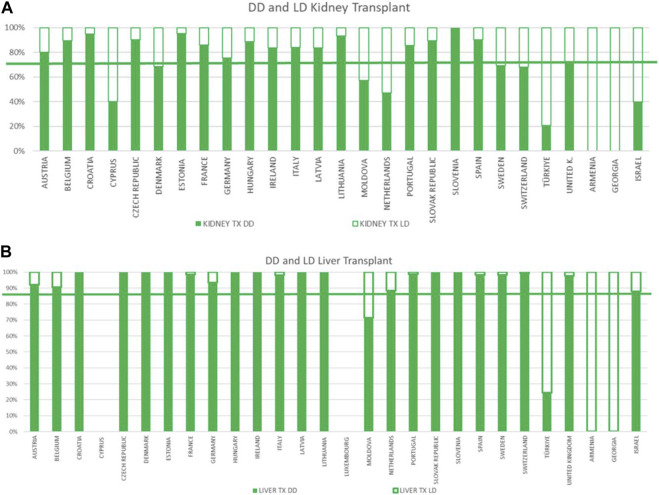
Kidney and liver deceased or living donors in CD-P-TO MS and OC. **(A)** Information regarding the kidney deceased and living donors; **(B)** information regarding the liver deceased and living donors.

LD transplantation was reported by 26 respondent countries [Figure 3]. In particular, there were 6,950 LKD and 1,383 LLD. As far as LKD, living donors accounted for only 30% of the transplanted kidneys, reaching the highest rate in Turkey (79% kidney transplants). Females were the most common donors, accounting for 58% of the donors, but received only 36% of the organs from LKD [Supplementary Figure S1]. As far as LLD, living donor lobe donation accounted for 17% of the liver transplants performed in Europe; it was performed in only 12 of the respondent countries and represented 76% of living liver transplants performed in Turkey. Females represented only 46% of LLD and received 42% of organs from LLD [Supplementary Figure S1]. The mean age of living donors according to sex and organ type was 49.3 years for males and 50 years for females LKD; and 47.1 years and 50 years respectively, for LLD.

### Recipient Information

**FIGURE 4:**
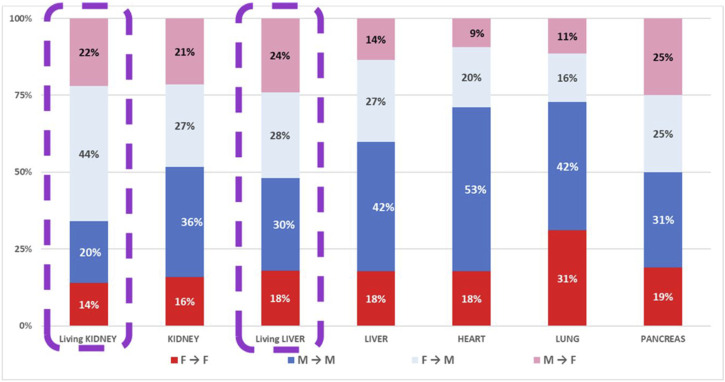
Recipients of organs from DD and LD according to sex pairs.

The sex pairs in the case of living or deceased donors are reported in Figure 4. Our data confirm that men received the majority of the organs from deceased donors. In particular, males received 63% of kidneys, 69% of livers, 73% of hearts, 58% of lungs, and 56% of pancreases.

Similarly, as far as organs from living donors, males received the majority of the kidneys (64%) and livers (58%) available.

### Sex Pairs and Post-Transplant Outcomes

**FIGURE 5:**
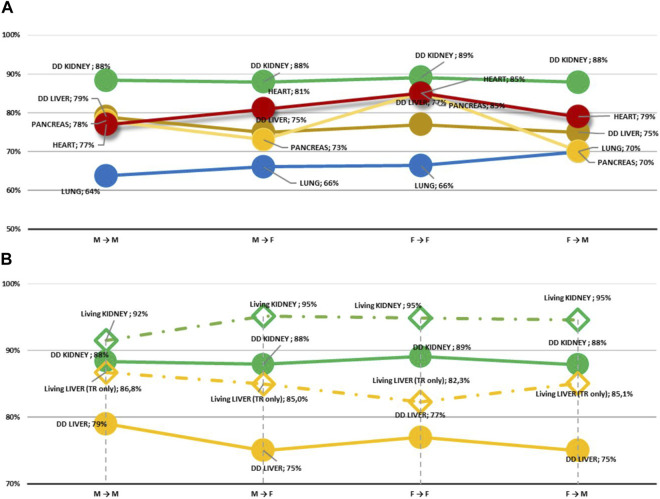
5-Year graft survival. **(A)** Recipients from DD according to sex pairs (all organs); **(B)** Kidney and liver recipients from DD and LD according to sex pairs.

Five-year graft survival according to sex pairs is reported in Figure 5. As expected, for each of the sex pairs organs from living donors had a better 5-year survival compared to the deceased counterpart. On the other hand, in our study 5-year graft survival (for organs either from deceased or living donors) did not differ when considering the different sex pair combinations.

### Relationship Between Living Donors and Recipients

**FIGURE 6:**
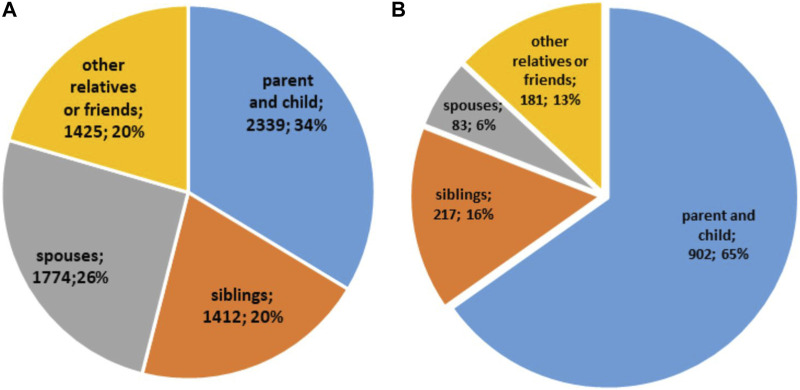
Relationship between living donors and recipients. **(A)** Relationship between LK donors and recipients; **(B)** Relationship between LL donors and recipients.

Out of a total number of 6,950 LKD, 2,339 (34%) organs were transplanted between parent and child; 1,412 (20%) between siblings; 1,774 (26%) between spouses; and 1,425 (20%) between other relatives or friends [Figure 6]. The relationship between LKDs and recipients in the responding countries is reported in Supplementary Figure S2. Kidneys from male living donors (42% of the kidneys from LKD) were transplanted into 49% of male recipients; in contrast, kidneys from female living donors (58% of the kidneys from LKD) were primarily transplanted into males (75%) [Supplementary Figure S3]. The relationship between LKD and recipients distributed according to sex pairs is reported in Supplementary Figure S4.

Out of a total number of 1,383 LLD, 902 (65%) organs were transplanted between parent and child; 217 (16%) between siblings; 83 (6%) between spouses and 181 (13%) between other relatives or friends [Figure 6]. The relationship between LLD and recipients in the responding countries is reported in Supplementary Figure S2. Livers donated from male donors (54% of the overall number of livers from LLD) were transplanted in 55% of cases into male recipients; in contrast, livers from female donors (46% of the overall number of livers from LLD) were primarily transplanted into male recipients (63% cases) [Supplementary Figure S3B]. The relationship between donors and recipients distributed according to sex pairs are reported in Supplementary Figure S4B.

## Discussion

Transplantation is the most effective therapeutic option for eligible patients diagnosed with advanced or end stage organ failure. Even though countries performing organ transplantation, make the treatment accessible to all eligible patients, irrespective of sex, age and race, it has been previously reported that, compared to men, women are disadvantaged with regards to both access to active transplant waiting lists and access to transplantation after waitlisting [13–17].

Indeed, women have lower access to transplantation. In many cases, this is the ultimate outcome of unbiased circumstances that include a higher burden of diseases treatable with transplantation among men [5, 6], organ size mismatch [18] or greater difficulties in finding an immunologically compatible donor in women due to prior immunizing events [4, 7].

Nonetheless, in the context of kidney transplantation, the choice of glomerular filtration rate (GFR) estimation methods for determining access to waiting lists remains a subject of debate. Indeed, both the MDRD and CKD-EPI equations, which are commonly used to estimate GFR, are creatinine-based and thus dependent on muscle mass; consequently, they tend to underestimate GFR in women, despite adjustments also in late chronic kidney disease (CKD) stages [19, 20]. In clinical practice, awareness of the systematic underestimation of GFR in women may create diagnostic uncertainty. This is particularly evident when evaluating borderline GFR values for transplant eligibility, where such bias can delay the initiation of the evaluation process, its completion, or final access to the waiting list—steps that are less frequent in female patients [21].

With regard to access to liver transplantation, the MELD score adopted in the United States two decades ago heavily weighted creatinine, ultimately generating a negative bias for women access to transplantation. In 2008, the MELD score was modified to incorporate serum sodium (MELD-Na). However, this update failed to mitigate—and may have even exacerbated—gender disparities, with women remaining 20% less likely to receive a transplant than men [22–24]. Furthermore, women experienced higher rates of waitlist mortality or delisting due to clinical deterioration [25]. This higher attrition rate may result, at least in part, from prolonged waiting times secondary to lower calculated MELD-Na scores.

Some reports suggest that socioeconomic or cultural factors may underlie the variation in access to transplantation between sex [3, 21, 26, 27]. Furthermore, even medical biases have been described as a possible explanation for the sex-related differences in access to this treatment [4, 28–30]. Sex-related imbalances in terms of both donation and access to transplantation have also been reported when transplantation takes place using organs from living donors [15].

To study whether sex-related aspects may contribute to a potential disparity in the transplantation activity in Europe, the CD-P-TO commissioned a further in depth analysis of organ donation and transplantation across the Council of Europe member states previously conducted in 2019 [1]. Our survey eventually enabled us to have access to data from 26 of the 39 Council of Europe Member States, ultimately covering an overall population of 527.4 million European citizens (UNFPA report [31]).

The data collected confirm that, irrespective of the organ considered, men still account for the majority of the patients awaiting transplantation.

To establish whether women on the WL wait longer than men, in this study we determined the median waiting time on the WL. Our data indicate that in the Council of Europe Member States, for all the organs except for hearts, women have a longer waiting time on the WL compared to men.

As far as organ donation is concerned, our data confirm that, as previously reported, male donors make up the majority of deceased donors (DBD or DCD) in Europe and females provide the majority of kidneys from LD. Interestingly, males make up the majority of living liver donors in the Council of Europe countries.

In light of earlier reports suggesting that sex pairs may affect graft survival [21, 32], the impact of sex combinations on 5-year graft survival was analysed for organs from both deceased and living donors. As expected, 5-year graft survival of organs from living donors had a better outcome than that of the deceased counterpart. However, in our study 5-year graft survival (for organs either from deceased or living donors) did not differ when considering the different sex pair combinations. While our data confirm recent findings suggesting that, *per se*, the patient sex may not represent a key determinant affecting transplant outcomes [33], we cannot rule out that the impact of the limited number of cases and the duration of the observation period in our study may explain the different findings compared to earlier reports [21, 32].

We also analysed the relationship between living donors and recipients. In Council of Europe countries, the parent-to-child pair represents the most frequent combination for both LKD and LLD. The greater involvement of parents in this case is not unexpected as having a child recipient and helping a family member are amongst the most motivating factors for considering living organ donation [34].

We acknowledge that our study presents several limitations. First, the analyses were conducted on data provided by only 26 of the 39 Council of Europe CD-P-TO Member States. Second, the data provided refer to an observation period of 1 year, a condition that may not accurately reflect the routine activities of some of the countries involved. Third, the use of the median waiting time on the WL as a means to establish whether women on the WL wait longer than men does not differentiate whether patients removal from the WL is due to access to transplantation, death on the waiting list or delisting due worsening condition. Fourth, because the data were collected in aggregate form, it was not possible to perform statistical inference analyses; therefore, only a descriptive analysis could be conducted, and differences could be evaluated solely in these terms. Nevertheless, the available data allowed us to gain an overall view of the situation. In this light, a follow up study in countries able to establish the cumulative incidence of access to transplantation for patients on the active waiting list will represent an important tool to increase our knowledge on the impact of sex on the access to transplantation for women on the active WL. Eventually, the data analysed refer to the year 2019, the last year that was not impacted by the COVID-19 pandemic. Still, this is the only set of data available that explore in detail the impact of sex on transplantation activities across the CD-P-TO Member States.

In summary, altogether the data collected from 26 Council of Europe Member States and 3 observer countries suggest that, whilst men represent the majority of patients on the organ transplant waiting lists and receive the largest number of organs from deceased or living donors, at this stage there is no evidence of unfair sex-related inequities in the transplantation activities conducted in Europe. Women provide the majority of kidneys from living donors whilst men are the major source of livers from living donors. Currently, however, we are still missing the sex-associated data regarding transplantation activities taking place in many of the Council of Europe Member States. In this light, the Newsletter Transplant has now broadened its annual data collection on organ transplantation activity to include data disaggregated by sex. In any case, this analysis provides a foundation for future in-depth studies aimed at understanding biological sex disparities in organ donation and transplantation. In this regard, the CD-P-TO will continue its monitoring activity on this highly relevant topic, possibly extending its commitment beyond sex to include gender related aspects. This should allow the CD-P-TO to uncover possibly unexpected imbalances and, where relevant, release recommendations that could act as a trigger for the adoption of new national policies.

## Data Availability

The raw data supporting the conclusions of this article will be made available by the authors, without undue reservation.

## Group Members of the European Committee on Organ Transplantation of the Council of Europe (CD-P-TO)

The CD-P-TO is the steering committee in charge of organ, tissue and cell donation and transplantation activities at the European Directorate for the Quality of Medicines and Healthcare of the Council of Europe. It actively promotes the non-commercialization of organ, tissue and cell donation, the fight against organ trafficking and the development of ethical, quality and safety standards in the field of organ, tissue and cell transplantation. Its activities include the collection of international data and monitoring of practices in Europe, the transfer of knowledge and expertise between organisations and experts through training and networking and the elaboration of reports, surveys, and recommendations. As of September 2025, the CD-P-TO was composed of 39 members (Albania, Austria, Belgium, Bosnia and Herzegovina, Bulgaria, Croatia, Cyprus, Czech Republic, Denmark, Estonia, Finland, France, Germany, Greece, Hungary, Iceland, Ireland, Italy, Lithuania, Latvia, Luxembourg, Malta, Montenegro, Netherlands, Republic of North Macedonia, Norway, Poland, Portugal, Romania, Serbia, Slovak Republic, Slovenia, Spain, Sweden, Switzerland, Republic of Moldova, Turkey, Ukraine, and United Kingdom) and 20 observers (Armenia, Canada, Georgia, Israel, United States, Council of Europe Committee on Bioethics, DTI Foundation, European Association of Tissue and Cell Banks, European Commission, European Eye Bank Association, European Society for Blood and Marrow Transplantation, European Society for Organ Transplantation, European Society of Human Reproduction and Embryology, Eurotransplant, Scandiatransplant, South-Europe Alliance for Transplants (SAT), The Transplantation Society, United Network for Organ Sharing (UNOS), World Health Organization (WHO), and World Marrow Donors Association).

## Supplementary Material

SUPPLEMENTARY TABLE 1 List of CDPTO Member States and Observer countries involved in the analysis.

SUPPLEMENTARY FIGURE S1 Sex combination of living donors and transplant recipients. **(A)** LKD and kidney transplant recipients; **(B)** LLD and liver transplant recipients.

SUPPLEMENTARY FIGURE S2 Relationship between living donors and recipients distributed per CD-P-TO MS and Observers Countries. **(A)** Relationship between living kidney donors and recipients distributed per country; **(B)** Relationship between living liver donors and recipients distributed per country.

SUPPLEMENTARY FIGURE S3 Destination of organs donated by M and F donors in CD-P-TO MS. **(A)** Living donor kidney transplantation (n=6.950); **(B)** Living donor liver transplantation (n = 1.383).

SUPPLEMENTARY FIGURE S4 Relationship between living kidney and liver donors and recipients according to sex pairs in CD-P-TO MS. **(A)** Relationship between living kidney donors and recipients according to sex pairs (%); **(B)** Relationship between living liver donors and recipients according to sex pairs (%).

SUPPLEMENTARY FILE S1 Gender aspects in transplantation: expanded disaggregated activity data collection and analysis (TO115).
