# Supplementary figures and images for: The Sex of Donor and Recipients in Solid Organ Transplantation: An in Depth Analysis Across the Council of Europe Member States
Source: Transpl Int. 2026 May 7;39:15711. doi: 10.3389/ti.2026.15711 (PMC13189984; doi:10.3389/ti.2026.15711)

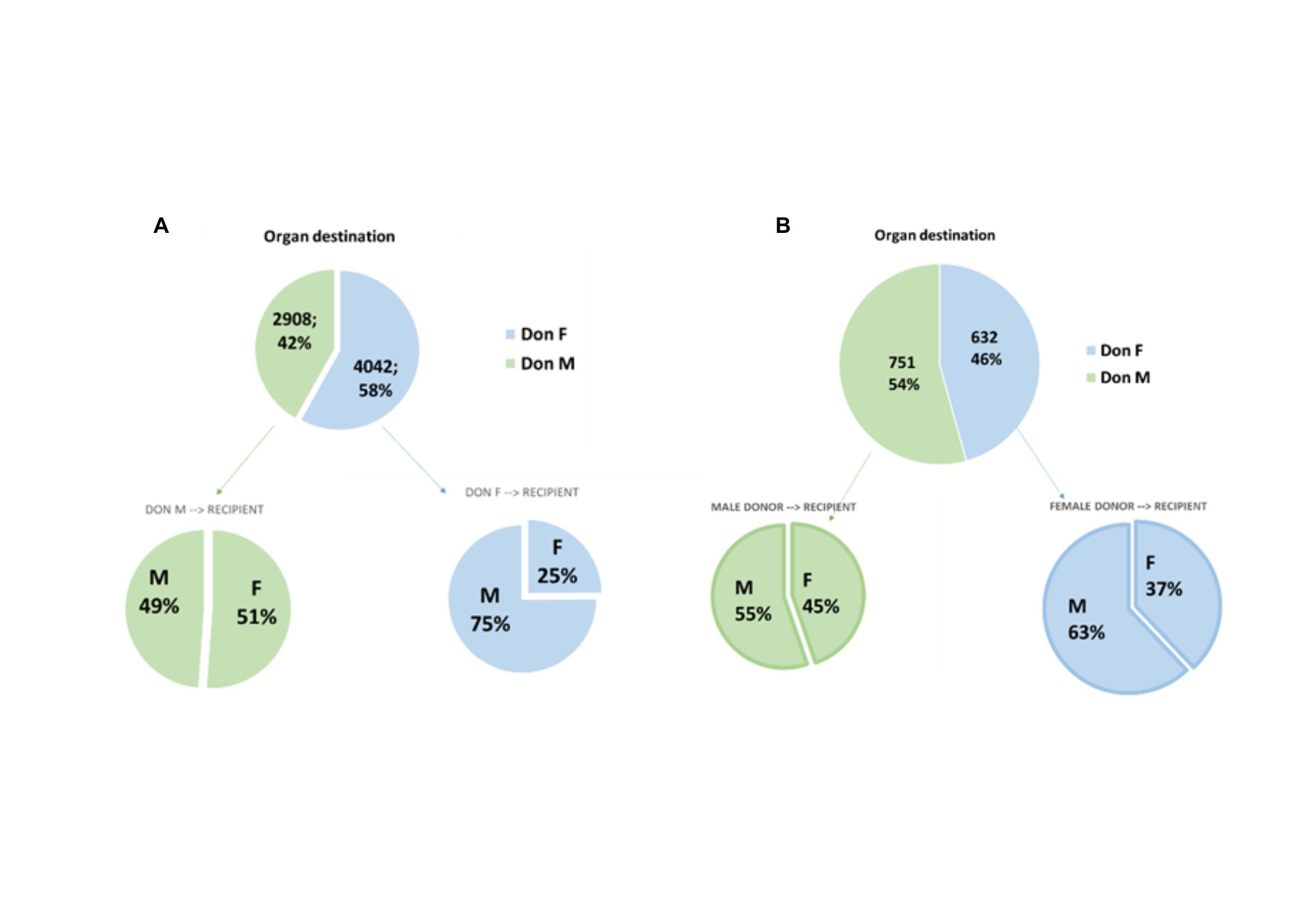

Supplement: Supplementary file 1 [file Image3.jpeg]

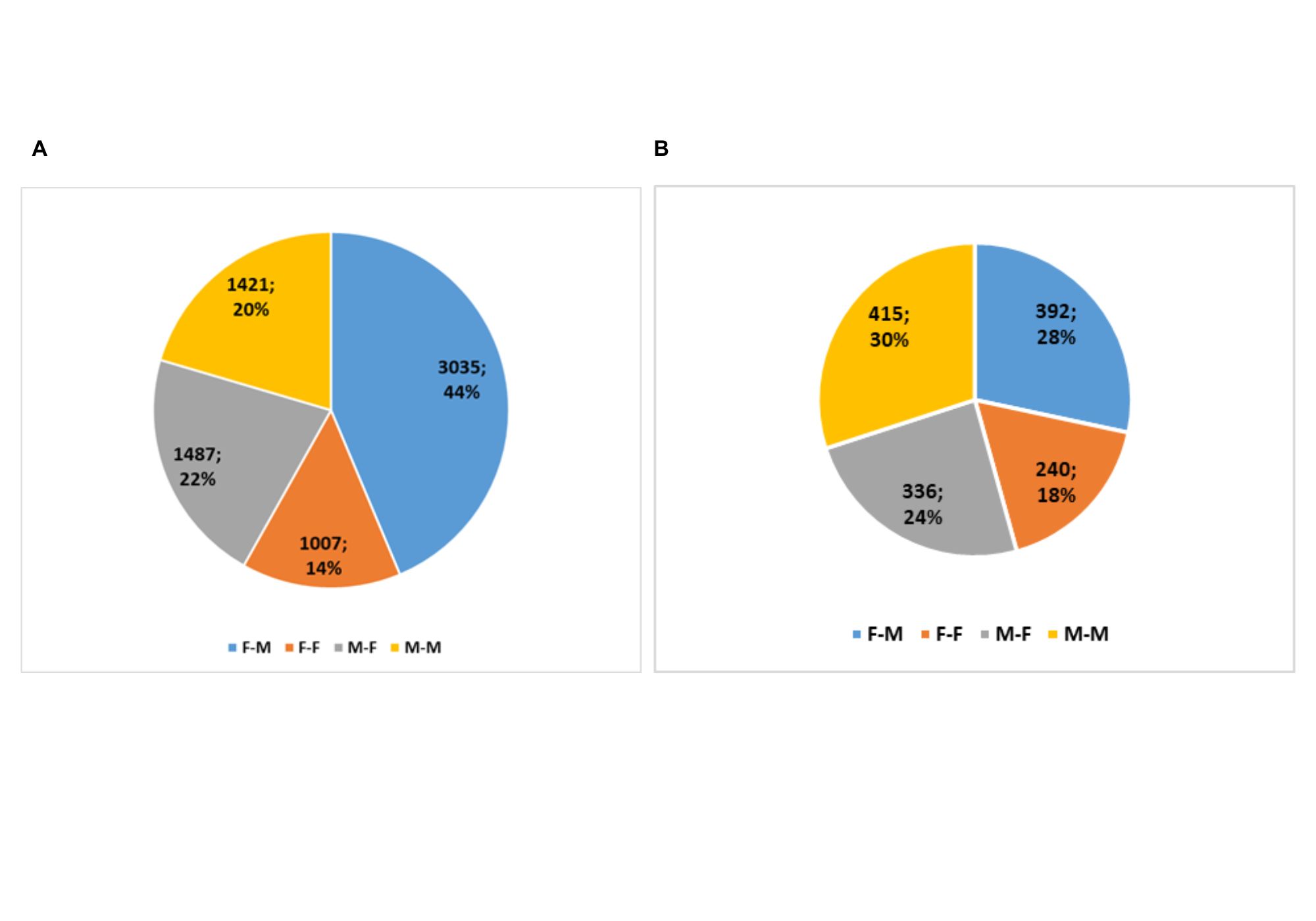

Supplement: Supplementary file 4 [file Image1.jpeg]

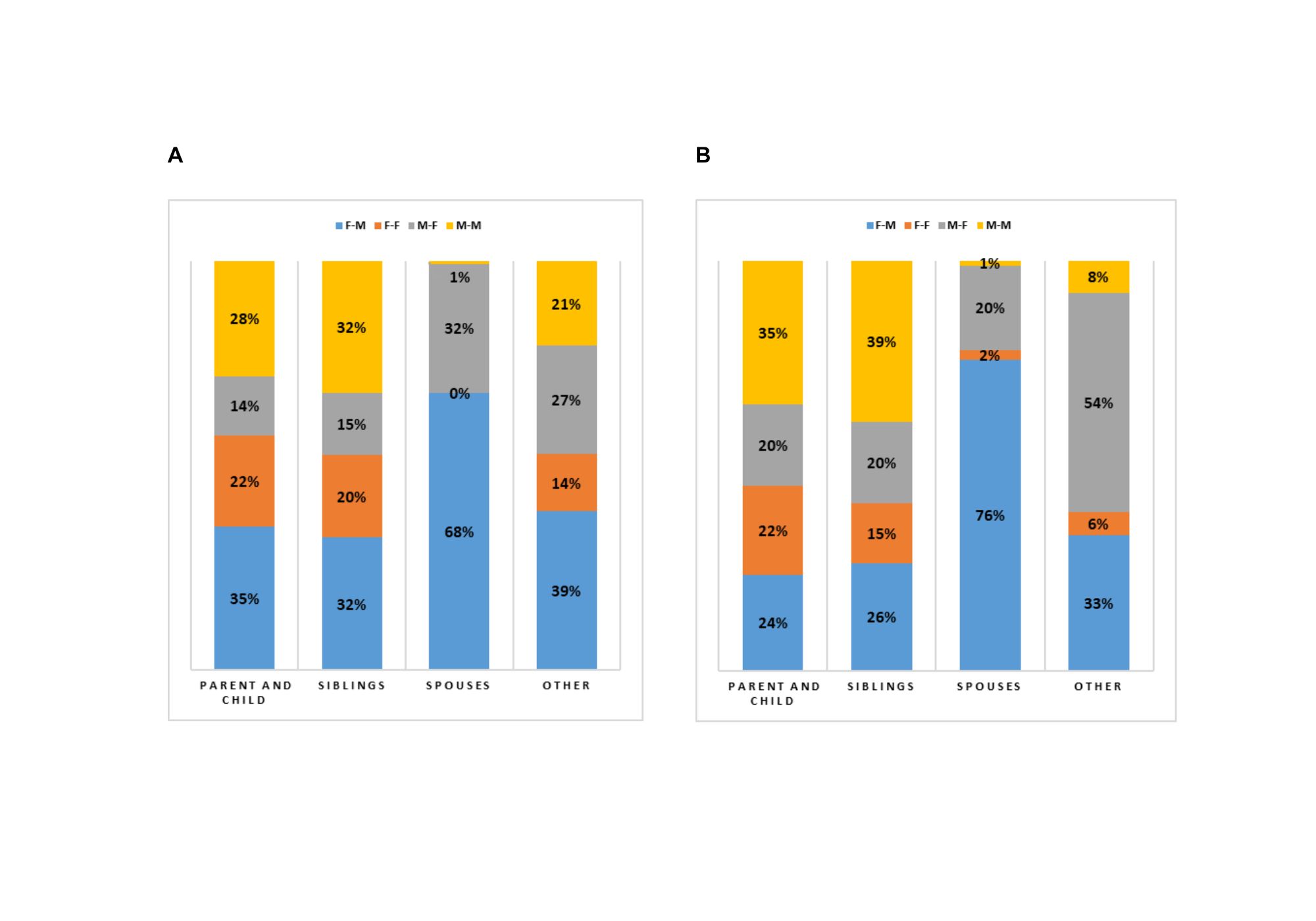

Supplement: Supplementary file 5 [file Image4.jpeg]

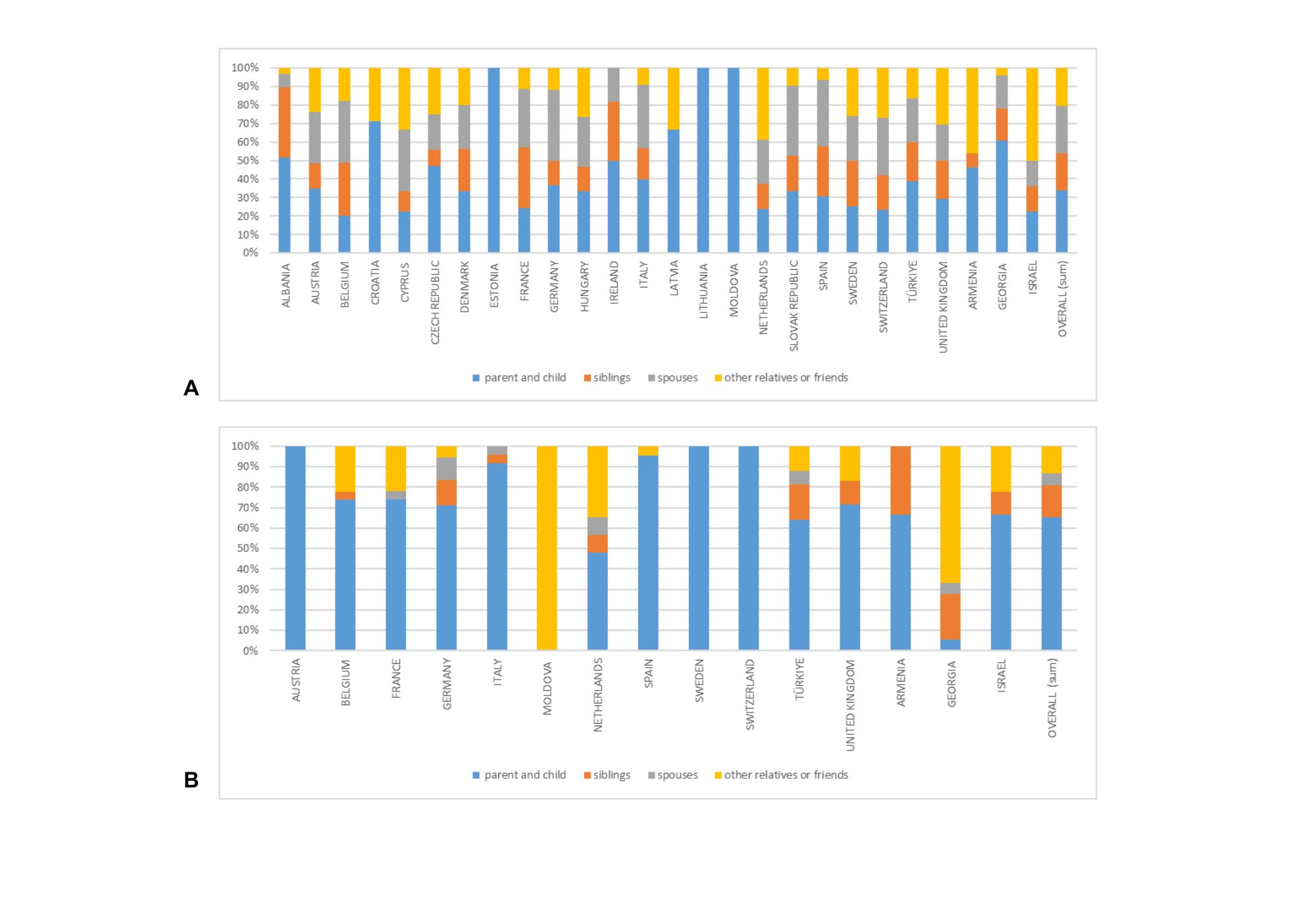

Supplement: Supplementary file 6 [file Image2.jpeg]
