# Supplementary material for: The Sex of Donor and Recipients in Solid Organ Transplantation: An in Depth Analysis Across the Council of Europe Member States
Source: Transpl Int. 2026 May 7;39:15711. doi: 10.3389/ti.2026.15711 (PMC13189984; doi:10.3389/ti.2026.15711)
Supplement: Supplementary file 2 [file Supplementaryfile1.docx]

# Gender aspects in transplantation: expanded disaggregated activity data collection and analysis (TO115)

SURVEY

# Your details

| Country |  |
| --- | --- |
| Name of the organization: |  |
| Job Title of the person completing the survey: |  |

**Contact details:**

| Name: |  |
| --- | --- |
| email address: |  |
| telephone number: |  |

# Section A: General information regarding the national organ transplantation activities for the year 2019

The general information regarding the national organ transplantation activities in your country for the year 2019 has already been collected and is available for the Newsletter Transplant.

# Section B: General information regarding the national organ transplantation waiting lists for the year 2019

Please note: total waiting time is calculated from day 1 of dialysis

| Overall, the number of patients waiting for an organ transplant in your country who were ever active on the waiting list along the year was: |  |
| --- | --- |

| In relation to **KIDNEY** transplantation: | | | |
| --- | --- | --- | --- |
| The information regarding the overall number of patients on the waiting list is already available. However, of these we would like to know: | | | |
| the number of males | |  | |
| the number of females | |  | |
| For patients listed in 2019: | years | | range, in years |
| the median waiting time for a **KIDNEY** transplant was: |  | |  |
| In particular: | years | | range, in years |
| the median waiting time for a male was: |  | |  |
| the median waiting time for a female was: |  | |  |
|  |  | |  |

| In relation to **HEART** transplantation: | | | |
| --- | --- | --- | --- |
| The information regarding the overall number of patients on the waiting list is already available. However, of these we would like to know: | | | |
| the number of males | |  | |
| the number of females | |  | |
| For patients listed in 2019: | years | | range, in years |
| the median waiting time for a **HEART** transplant was: |  | |  |
| In particular: | years | | range, in years |
| the median waiting time for a male was: |  | |  |
| the median waiting time for a female was: |  | |  |
|  |  | |  |

| In relation to **LUNG** transplantation: | | | |
| --- | --- | --- | --- |
| The information regarding the overall number of patients on the waiting list is already available. However, of these we would like to know: | | | |
| the number of males | |  | |
| the number of females | |  | |
| For patients listed in 2019: | years | | range, in years |
| the median waiting time for a **LUNG** transplant was: |  | |  |
| In particular: | years | | range, in years |
| the median waiting time for a male was: |  | |  |
| the median waiting time for a female was: |  | |  |
|  |  | |  |

| In relation to **LIVER** transplantation: | | | |
| --- | --- | --- | --- |
| The information regarding the overall number of patients on the waiting list is already available. However, of these we would like to know: | | | |
| the number of males | |  | |
| the number of females | |  | |
| For patients listed in 2019: | years | | range, in years |
| the median waiting time for a **LIVER** transplant was: |  | |  |
| In particular: | years | | range, in years |
| the median waiting time for a male was: |  | |  |
| the median waiting time for a female was: |  | |  |
|  |  | |  |

| In relation to **PANCREAS** transplantation: | | | |
| --- | --- | --- | --- |
| The information regarding the overall number of patients on the waiting list is already available. However, of these we would like to know: | | | |
| the number of males | |  | |
| the number of females | |  | |
| For patients listed in 2019: | years | | range, in years |
| the median waiting time for a **PANCREAS** transplant was: |  | |  |
| In particular: | years | | range, in years |
| the median waiting time for a male was: |  | |  |
| the median waiting time for a female was: |  | |  |
|  |  | |  |

# Section C: Information regarding the donors

## Section C.1: DCD

| As far as the age of DCD category II / Witnessed cardiac arrest (uncontrolled): | | | | |
| --- | --- | --- | --- | --- |
|  | Years (±) | | Range in years | |
| the mean age of male DCD II was: |  |  |  |  |
| the mean age of female DCD II was: |  |  |  |  |

| As far as the age of DCD category III / Withdrawal of life-sustaining therapy (controlled): | | | | |
| --- | --- | --- | --- | --- |
|  | Years (±) | | Range in years | |
| the mean age of male DCD III was: |  |  |  |  |
| the mean age of female DCD III was: |  |  |  |  |

| As far as the age of DCD category IV / Cardiac arrest while brain dead: | | | | |
| --- | --- | --- | --- | --- |
|  | Years (±) | | Range in years | |
| the mean age of male DCD IV was: |  |  |  |  |
| the mean age of female DCD IV was: |  |  |  |  |

## Section C.2: DBD

| As far as the age of DBD: | | | | |
| --- | --- | --- | --- | --- |
|  | Years (±) | | Range in years | |
| the mean age of male DBD was: |  |  |  |  |
| the mean age of female DBD was: |  |  |  |  |

## Section C.3: LD

### Section C.3.1: Kidney LD

| As far as the age of the kidney LD: | | | | |
| --- | --- | --- | --- | --- |
|  | Years (±) | | Range in years | |
| the mean age of male KLD was: |  |  |  |  |
| the mean age of female KLD was: |  |  |  |  |

### Section C.3.2: Liver LD

| As far as the age of the liver LD: | | | | |
| --- | --- | --- | --- | --- |
|  | Years (±) | | Range in years | |
| the mean age of male LLD was: |  |  |  |  |
| the mean age of female LLD was: |  |  |  |  |

# Section D: Information regarding the recipients

## Section D.1: recipients of organs from deceased donors (DCD + DBD)

### Section D.1.1: **Kidney**

| **Kidney** recipients from deceased donors | |
| --- | --- |
| the number of male recipients was: |  |
| the number of female recipients was: |  |

### Section D.1.2: **Heart**

| **Heart** recipients from deceased donors | |
| --- | --- |
| the number of male recipients was: |  |
| the number of female recipients was: |  |

### Section D.1.3: **Lung**

| **Lung** recipients from deceased donors | |
| --- | --- |
| the number of male recipients was: |  |
| the number of female recipients was: |  |

### Section D.1.4: **Liver**

| **Liver** recipients from deceased donors | |
| --- | --- |
| the number of male recipients was: |  |
| the number of female recipients was: |  |

### Section D.1.5: **Pancreas**

| **Pancreas** recipients from deceased donors | |
| --- | --- |
| the number of male recipients was: |  |
| the number of female recipients was: |  |

## Section D.2: recipients of organs from LD

The information regarding the recipients of organs from living donors in your country for the year 2019 has already been collected and is available for the Newsletter Transplant.

# Section E: transplantation and sex pairs

## Section E.1: sex pairs in organ transplantation from deceased donors

### **Section E.1.1:** sex pairs in **kidney** transplantation from deceased donors

| M → M |  |
| --- | --- |
| M → F |  |
| F → F |  |
| F → M |  |

### Five years survival according to sex pairs

| M → M |  |
| --- | --- |
| M → F |  |
| F → F |  |
| F → M |  |

### **Section E.1.2:** sex pairs in **heart** transplantation from deceased donors

| M → M |  |
| --- | --- |
| M → F |  |
| F → F |  |
| F → M |  |

### Five years survival according to sex pairs

| M → M |  |
| --- | --- |
| M → F |  |
| F → F |  |
| F → M |  |

### **Section E.1.3:** sex pairs in **lung** transplantation from deceased donors

| M → M |  |
| --- | --- |
| M → F |  |
| F → F |  |
| F → M |  |

### Five years survival according to sex pairs

| M → M |  |
| --- | --- |
| M → F |  |
| F → F |  |
| F → M |  |

### **Section E.1.4:** sex pairs in **liver** transplantation from deceased donors

| M → M |  |
| --- | --- |
| M → F |  |
| F → F |  |
| F → M |  |

### Five years survival according to sex pairs

| M → M |  |
| --- | --- |
| M → F |  |
| F → F |  |
| F → M |  |

### **Section E.1.5:** sex pairs in **pancreas** transplantation from deceased donors

| M → M |  |
| --- | --- |
| M → F |  |
| F → F |  |
| F → M |  |

### Five years survival according to sex pairs

| M → M |  |
| --- | --- |
| M → F |  |
| F → F |  |
| F → M |  |

## Section E2: Sex pairs in organ transplantation from LD

### **Section E.2.1:** sex pairs in **kidney** transplantation from LD

| M → M |  |
| --- | --- |
| M → F |  |
| F → F |  |
| F → M |  |

### Five years survival according to sex pairs

| M → M |  |
| --- | --- |
| M → F |  |
| F → F |  |
| F → M |  |

### **Section E.2.2:** sex pairs in **liver** transplantation from LD

| M → M |  |
| --- | --- |
| M → F |  |
| F → F |  |
| F → M |  |

### Five years survival according to sex pairs

| M → M |  |
| --- | --- |
| M → F |  |
| F → F |  |
| F → M |  |

# Section F: relationship between living donors and recipients

## Section F.1: relationship between kidney living donors and recipients

### **Section F.1.1:** in the case of KLD between parent and child, the combinations were the following:

|  | n. of cases |
| --- | --- |
| mother → son: |  |
| mother → daughter |  |
| father → son |  |
| father → daughter |  |
|  | n. of cases |
| son → mother |  |
| son → father |  |
| daughter → mother |  |
| daughter → father |  |

### **Section F.1.2:** in the case of KLD between siblings, the combinations were:

|  | n. of cases |
| --- | --- |
| brother → brother |  |
| brother → sister |  |
| sister → brother |  |
| sister → sister |  |

### **Section F.1.3:** in the case of KLD between spouses, the combinations were:

|  | n. of cases |
| --- | --- |
| husband → wife |  |
| wife → husband |  |
| husband → husband |  |
| wife → wife |  |

### **Section F.1.4:** in the case of KLD between other relatives or friends, the combinations were:

|  | n. of cases |
| --- | --- |
| male → female |  |
| female → male |  |
| female → female |  |
| male → male |  |

## Section F.2: relationship between liver living donors and recipients

### **Section F.2.1:** in the case of LiLD between parent and child, the combinations were the following:

|  | n. of cases |
| --- | --- |
| mother → son: |  |
| mother → daughter |  |
| father → son |  |
| father → daughter |  |
|  | n. of cases |
| son → mother |  |
| son → father |  |
| daughter → mother |  |
| daughter → father |  |

### **Section F.2.2:** in the case of LiLD between siblings, the combinations were:

|  | n. of cases |
| --- | --- |
| brother → brother |  |
| brother → sister |  |
| sister → brother |  |
| sister → sister |  |

### **Section F.2.3:** in the case of LiLD between spouses, the combinations were:

|  | n. of cases |
| --- | --- |
| husband → wife |  |
| wife → husband |  |
| husband → husband |  |
| wife → wife |  |

### **Section F.2.4:** in the case of LiLD between other relatives and friends, the combinations were:

|  | n. of cases |
| --- | --- |
| male → female |  |
| female → male |  |
| female → female |  |
| male → male |  |

# Section G: follow-up questionnaire on outcome data based on donor-recipient sex combinations

Would you be willing and able to provide outcome data based on donor-recipient sex combinations: YES  NO
