# Supplementary material for: The Sex of Donor and Recipients in Solid Organ Transplantation: An in Depth Analysis Across the Council of Europe Member States
Source: Transpl Int. 2026 May 7;39:15711. doi: 10.3389/ti.2026.15711 (PMC13189984; doi:10.3389/ti.2026.15711)
Supplement: Supplementary file 3 [file Table1.docx]

**SUPPLEMENTARY TABLE 1**

| **CD-P-TO Member States** | |
| --- | --- |
| ALBANIA | Ministry of Health and Social Protection, UHC “Mother Teresa” |
| AUSTRIA | Federal Ministry of Labour, Social Affairs, Health and Consumer Protection |
| BELGIUM | Agence Fédérale du Médicament et des Produits de Santé |
| CROATIA | Ministry of Health of the Republic of Croatia |
| CYPRUS | Nicosia General Hospital |
| CZECH REPUBLIC | Transplant Coordinating Center |
| DENMARK | Danish Patient Safety Authority |
| ESTONIA | Tartu University Hospital |
| FRANCE | Agence de la Biomédecine |
| GERMANY | Deutsche Stiftung Organtransplantation |
| HUNGARY | Hungarian National Blood and Transfusion Service |
| IRELAND | Organ Donation Tranplant Ireland |
| ITALY | Italian National Transplant Centre-Italian National Institute of Health |
| LATVIA | Children Clinical University Hospital |
| LITHUANIA | National Transplant Bureau |
| LUXEMBOURG | Ministry of Health |
| MOLDOVA | Transplant Agency of Moldova |
| NETHERLANDS | Dutch Transplantation Foundation |
| PORTUGAL | Portuguese Institute for Blood and Transplantation |
| SLOVAK REPUBLIC | University Hospital Martin |
| SLOVENIA | Slovenija Transplant |
| SPAIN | Organización Nacional de Trasplantes |
| SWEDEN | The Swedish National Board of Health and Welfare |
| SWITZERLAND | Office Fédéral de la Santé Publique |
| TÜRKIYE | Istanbul Bilim University |
| UNITED KINGDOM | NHS Blood and Transplant |
| **Observer Countries** | |
| ARMENIA | "Arabkir" medical centre |
| GEORGIA | Georgian Association of Transplantologists |
| ISRAEL | Israel National Transplat Center |
